# Mesenchymal Stem Cell Therapy for Aging Frailty

**DOI:** 10.3389/fnut.2018.00108

**Published:** 2018-11-15

**Authors:** Ivonne Hernandez Schulman, Wayne Balkan, Joshua M. Hare

**Affiliations:** ^1^Interdisciplinary Stem Cell Institute, University of Miami Miller School of Medicine, Miami, FL, United States; ^2^Katz Family Division of Nephrology and Hypertension, University of Miami Miller School of Medicine, Miami, FL, United States

**Keywords:** cell transplantation, regenerative medicine, inflammation, immunosenescence, geriatrics

## Abstract

Chronic diseases and degenerative conditions are strongly linked with the geriatric syndrome of frailty and account for a disproportionate percentage of the health care budget. Frailty increases the risk of falls, hospitalization, institutionalization, disability, and death. By definition, frailty syndrome is characterized by declines in lean body mass, strength, endurance, balance, gait speed, activity and energy levels, and organ physiologic reserve. Collectively, these changes lead to the loss of homeostasis and capability to withstand stressors and resulting vulnerabilities. There is a strong link between frailty, inflammation, and the impaired ability to repair tissue injury due to decreases in endogenous stem cell production. Although exercise and nutritional supplementation provide benefit to frail patients, there are currently no specific therapies for frailty. Bone marrow-derived allogeneic mesenchymal stem cells (MSCs) provide therapeutic benefits in heart failure patients irrespective of age. MSCs contribute to cellular repair and tissue regeneration through their multilineage differentiation capacity, immunomodulatory, and anti-inflammatory effects, homing and migratory capacity to injury sites, and stimulatory effect on endogenous tissue progenitors. The advantages of using MSCs as a therapeutic strategy include standardization of isolation and culture expansion techniques and safety in allogeneic transplantation. Based on this evidence, we performed a randomized, double-blinded, dose-finding study in elderly, frail individuals and showed that intravenously delivered allogeneic MSCs are safe and produce significant improvements in physical performance measures and inflammatory biomarkers. We thus propose that frailty can be treated and the link between frailty and chronic inflammation offers a potential therapeutic target, addressable by cell therapy.

## Definition and epidemiology of frailty

Frailty has been clinically defined as “a state of increased vulnerability resulting from aging-associated decline in reserve and function across multiple organ systems such that the ability to cope with everyday or acute stressors is compromised” (1). Central to this geriatric medical syndrome is the notion that it has multiple causes and contributors that lead to the characteristic decreases in strength, endurance, activity, energy levels, and physiologic function, which increase the susceptibility to dependency and death (2, 3). Of note, although frailty is not characterized as a disability, it does increase the risk of disability in affected individuals (3–5). Moreover, there is a close link between a patient's health and frailty (6, 7). These patients tend to show a greater risk of frailty when there are other comorbidities affecting their physical and psychological well-being, such as cardiovascular disease, diabetes, high blood pressure, cancer, or cognitive impairment (8). The main clinical presentations of frailty are falls, which are a result of impaired balance, gait, and awareness, fluctuating disability with independent and dependent days, and other non-specific signs and symptoms, such as unexplained weight loss, infections, and extreme fatigue.

Several instruments have been developed to assess frailty. These can be categorized as the unidimensional or phenotypic model, based on the physical or biological dimension, and multidimensional or cumulative deficit models, centered on the links between the physical, psychological, and social realms, all of which have been well validated (1, 8). The unidimensional or phenotypic model was first operationally defined by Fried et al. (9) and was used to develop the Cardiovascular Health Study (CHS) Index, commonly referred to as the “Physical Frailty Phenotype” (10). Using this model, frailty is defined as having three out of five phenotypic criteria indicating “compromised energetics”: weak grip strength, low energy levels or self-reported exhaustion, slow gait speed, low physical activity (low energy expenditure), and/or unintentional weight loss (9). On the other hand, the Canadian Study of Health and Aging (CSHA) frailty index was developed using the cumulative deficit model (11, 12). The CSHA frailty index measures several age-associated health deficits (13), and is computed by counting the number of health deficits and dividing this number by the total number of health questions tested (12), with a score of 1 being the maximum index and indicating the poorest prognosis. Indeed, an index >0.7 is associated with a high risk of mortality (14). This index has been simplified for use in the outpatient clinic setting as the CSHA “clinical frailty scale” (15). This 7-point rapid screening tool, consisting of 7 variables ranging from fit to complete functional dependence, highly correlates with the frailty index. The maximum in this scale is a score of 7, indicating “severe frailty” (16).

A number of other instruments to assess frailty have been developed and validated, including the “FRAIL (Fatigue, Resistance, Ambulation, Illnesses, Loss of weight)” frailty scale by the International Academy of Nutrition and Aging (17), the “Study of Osteoporotic Fractures (SOF)” frailty scale (18), the “Frailty Instrument for Primary Care of the Survey of Health, the Aging and Retirement in Europe (SHARE-FI)” scale (19), and the “Groningen Frailty Indicator” (20). The FRAIL and SOF, for example, predict new disability at 3 and 9 years of follow up and the FRAIL predicts 9-year mortality in an African American population (21). A multidimensional instrument based on a “structural questionnaire” is the “Tilburg Frailty Indicator” (TFI) (22). This instrument is made up of 10 questions on determinants of frailty that include demographics and other lifestyle questions, as well as, 15 frailty elements arranged according to physical, psychological, and social aspects. The total score of the TFI ranges from 0 to 15, with frailty ascertained if the total score is 5 or greater.

In view of the aging population worldwide, there is a growing medical and scientific interest in the accurate diagnosis and treatment of frailty. Despite multinational efforts to reach an agreement on the definition of frailty and how to assess it with a simple and easily accessible tool, no consensus has been reached, as evidenced by the various definitions and multiple assessment tools being currently used in the literature. However, an agreement has been reached broadly defining frailty as a clinical syndrome characterized by increased vulnerability to stressors that leads to functional impairments and adverse health outcomes (2). This definition is considered to be useful in primary care assessments. Moreover, these functional impairments and health outcomes may be preventable or treatable by pharmacologic or non-pharmacologic interventions.

With regards to the prevalence of frailty in community dwelling individuals over the age of 65 in the United States, it was estimated at 7–12% using the frailty criteria validated in the CHS (10). Moreover, frailty prevalence increased with age from 3.9 to 25% in the 65–74 and over 85 age groups, respectively. Frailty prevalence was also found to be greater in women than men (8 vs. 5%). Ethnic differences in frailty prevalence were noted, with black Americans more likely to be frail than white Americans (13 vs. 6%) and Mexican Americans similar to Caucasians 7.8% (23), based on the Hispanic Established Populations Epidemiologic Studies of the Elderly. Compared to frailty, pre-frailty has a much greater prevalence, ranging between 35 and 50% in adults aged 65 or older. Pre-frailty is considered to be present in patients exhibiting one or two of the phenotypic criteria described in CHS and is reportedly more common in women than in men, just like frailty (9). There is also an association between pre-frailty and lower educational level and socio-economic status (24, 25). Despite the higher accumulation of deficits in women than in men of the same age, men exhibit a higher risk of mortality even though this accumulation is associated with mortality in both genders (26–28). Importantly, comorbidities, especially cardiovascular, pulmonary, musculoskeletal, neurologic, and psychiatric, are more prevalent in pre-frail compared to non-frail persons (24, 25, 29).

## Frailty and cardiovascular performance

The prevalence of cardiovascular disease (CVD) increases substantially in individuals 65 years of age and over, and especially in individuals aged 80 and over (30). Not surprisingly, increased CVD prevalence is linked with increased prevalence and incidence of frailty, as shown in a meta-analysis of 54,250 elderly patients without frailty at baseline (31).

The aging cardiovascular system has some very specific phenotypic alterations (9, 30, 32). These include aortic stiffness due to increased collagen and decreased elastin, endothelial dysfunction, left ventricular hypertrophy, and a diminution in exercise induced increase in ejection fraction. These characteristic abnormalities are hypothesized to contribute to specific symptoms of the frailty syndrome and to increase the morbidity and mortality from CVD in elderly individuals (7, 10, 30). Several studies document the increased risk for mortality in frail elderly patients with cardiovascular events such as non-ST-segment elevation myocardial infarction (NSTEMI) (3, 7). Frail individuals have increased disease burden and therefore more prolonged recuperation vs. a non-frail subject (2, 8, 10). There are additional associations between frailty and other cardiovascular diagnoses including angina, myocardial infarction, hypertension, heart failure with reduced ejection (HFrEF), heart failure with preserved ejection fraction (HFpEF), and stroke (10, 30). Gait speed is one symptom of frailty that is linked with increased cardiovascular events and mortality, specifically in ST elevation myocardial infarction patients (33, 34). Importantly, frailty presents a major challenge to the ability of CVD patients to undergo surgery and other medical interventions successfully, thus affecting outcomes (30).

## Role of inflammation in aging and frailty

“Inflammaging” is a term that has been used to depict the particular molecular and cellular inter-related events that promote the process of aging (35). With aging, there is a continuous accumulation of damaged macromolecules and cells, generation of toxic metabolites and microbial byproducts, and development of cellular senescence and immunosenescence (36, 37). Not only does inflammaging accelerate the aging process, it is linked with and accelerates the diseases associated with aging, including cardiovascular diseases, cognitive, and neurologic impairments, cancer, and degenerative joint disease. Importantly, the increased susceptibility to disease and death is a result of these molecular inflammation-related changes in physiological systems. As such, measuring the molecules or biomarkers that mediate inflammation has become a useful tool to assess the aging process (37). For instance, there is evidence that circulating levels of pro-inflammatory cytokines increase during aging. High levels of TNF-α, interleukin-6 (IL-6), and C-reactive protein (CRP), even in elderly populations considered healthy, are independent predictors of mortality (38). This same inflammatory response underlies the tissue damage linked to various age-related chronic diseases (39). Indeed, a multitude of studies have now reliably demonstrated that chronically high levels of pro-inflammatory biomarkers do predict risk of morbidity and mortality in the elderly population (37).

Frailty involves aging-related decreases in organ physiologic reserve, leading to impaired ability to withstand stressors and resulting in increased vulnerability to disease. Frail patients manifest disturbances in the hematologic and inflammatory systems, which seem to be at the core of this geriatric syndrome (37, 40). For instance, frail patients have elevated levels of fibrinogen, IL-6, factor VIII, D-dimer, and CRP compared to non-frail patients (32, 41). Studies also report reduced hemoglobin, high leukocytes, elevated TNF-α, and low vitamin D as biomarkers of frailty. Importantly, the inflammatory cytokine IL-6 strongly correlates with the frailty phenotype and with unfavorable health outcomes (32, 42–44). Of note, among frail subjects, women exhibit higher concentrations of inflammatory and coagulation factors than men (41).

CRP is an example of one biomarker that has a higher concentration in women experiencing symptoms of frailty. Differential white blood cell counts, on the other hand, similarly predict frailty risk in men and women. Although there is still insufficient data to show which markers specifically affect men or women, dysregulated inflammation is a considerable key physiological marker in correlation with the frailty syndrome in both genders. It is of interest to note that the strong correlation between frailty and inflammatory and hematologic biomarkers is remarkably similar to the strong correlation between CVD and these same biomarkers, supporting the notion that frailty and CVD are clinically interrelated (37). Importantly, frail individuals also have characteristic declines in cardiovascular reserve, as described above, which may in turn contribute to the symptoms of the syndrome.

Substantial evidence has shown that chronic inflammation underlies the syndrome of aging frailty, leading to impairments in mobility and gait, sarcopenia, osteopenia, and decreased strength. High levels of circulating IL-6 correlate with the development of mobility disability (45) and high levels of IL-6 and TNF-α, either alone or together, are linked with decreased muscle mass and strength, increasing the susceptibility to sarcopenia (46, 47). Moreover, high levels of IL-6 and CRP are independently associated with decreases in physical performance and strength in the elderly (48, 49). The Women's Health and Aging Study (WHAS) demonstrated that elevated IL-6 levels in older women were associated with a greater decline in the ability to walk and a greater risk of acquiring physical disabilities (50). The MacArthur Studies of Successful Aging also found an association between decreased walking speed and grip strength and elevated levels of IL-6 and CRP (51). With regards to all-cause mortality predictors, elevated systemic IL-6 levels correlate strongly with various causes of death, as well as, with mortality in the near future (52, 53). A strong correlation is also present between CRP and early mortality and TNF-α and mortality among the elderly (54, 55). Since these inflammatory biomarkers are not specifically indicative of a particular disease or cause of mortality, these increases in systemic inflammation are thought to reflect a fundamental aspect of the aging process (37).

Chronic inflammation also causes remodeling of the immune system, which diminishes immune responses and contributes to increased mortality in subjects over 60 years of age with frailty (32, 42–44). Immunologic remodeling, in turn, is an important pathophysiologic contributor to frailty in older humans (56, 57). This functional impairment in cell-mediated and humoral immunity in frailty is well documented (58–60) and leads to an increased vulnerability to infectious diseases (40). However, the level of inflammation that affects individuals with frailty is “low-grade,” for example, TNF-α levels range between 1.5 and 1.68 pg/ml (61). In this regard, it has been proposed that the chronicity of the inflammation causes more harm than the absolute level at any given time (62). This elevation in systemic TNF-α levels increases intracellular TNF-α in B cells, which causes a shift in B cell subsets producing an increased percentage of exhausted B cells and decreased switch memory B cells, thereby impairing B cell function (63, 64). In aging and frailty, T cell activity is also impaired, and can be assessed by a decrease in the ratio of CD4:CD8 cells (65). Together, these processes promote an immune cell refractory state where both T and B cell responses to *de novo* antigens and vaccines are diminished (66).

As there is no cure for aging or frailty, the therapeutic strategy is on developing approaches to lessen or at least regulate the effects of chronic inflammation on aging, with the goal to promote a healthier aging process. It is believed that frailty can ultimately be prevented or attenuated, and the link between frailty and inflammation offers a potential therapeutic target.

## Endogenous stem cells in frailty

An individual's endogenous stem cell production and function decreases with age and this decrease likely contributes to reduced ability to regenerate and repair organs and tissues (67–69). For instance, there is evidence that as mesenchymal stem cells (MSCs) undergo senescence, their multilineage differentiation and homing capacity and immunomodulatory and wound healing properties gradually disappear (69, 70). These aging-related declines may be due to intrinsic stem cell aging, for example there is evidence that aging induces a “quiescence-to-senescence switch” (71) in stem cells, and aging-related changes in extracellular matrix components and the stem cell niches in tissues (68, 72, 73). Collectively, these aging-related changes reduce stem cell self-renewal, maintenance and regenerative potential. With regard to frailty, altered and dysfunctional stem cell niches have been implicated in frailty syndrome (74, 75). As such, it has been proposed that a regenerative medicine therapeutic approach has the potential to improve or reverse the signs and symptoms of frailty (32, 70), as further discussed below.

## Mesenchymal stem cells as a therapeutic strategy for frailty

Medical advances and a more health aware society have contributed to a longer living population. However, as the population ages, the growing number of frail elderly patients will continue to increase the demand for healthcare services. Therefore, novel medical therapies for frailty are under investigation to address this unmet need amongst the elderly population. Although certain diets, especially the Mediterranean diet (76, 77), nutritional supplements (78), hormonal supplements (79), and exercise regimes (80, 81) have been shown independently or in combination (82) to improve the signs and symptoms of frailty (8), there is currently no specific medical therapy available to prevent or treat the frailty syndrome.

**Figure 1:**
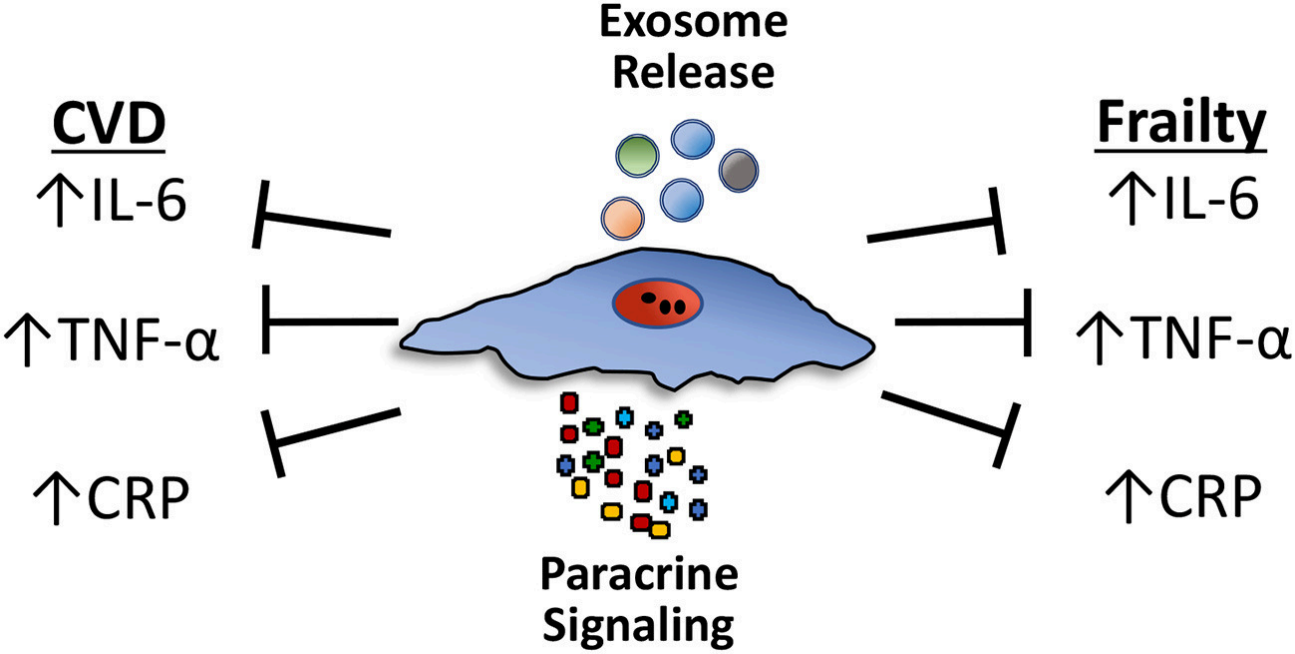
Circulating levels of proinflammatory cytokines, particularly TNF-α, interleukin-6 (IL-6), and C-reactive protein (CRP), increase during aging and are independent predictors of mortality in frail patients. These same pro-inflammatory cytokines are elevated and underlie the tissue damage linked to various age-related chronic diseases, particularly cardiovascular diseases (CVD). Mesenchymal stem cells (MSCs) reduce the expression of proinflammatory cytokines, including TNF-α, IL-6, and CRP, in both CVD and frailty syndrome through paracrine effects or via exosomes. Paracrine effects involve the secretion of a multitude of individual growth factors and cytokines. Exosomes are small extracellular vesicles that contain proteins, peptides and microRNAs (miRNAs).

There are specific features of the frailty syndrome that support a potential role of MSCs to ameliorate or improve frailty. MSCs are drawn to sites of injury, where they act to reduce inflammation and promote cellular repair (83). Notably, MSCs improve cardiovascular outcomes in patients with acute myocardial infarction (84), as well as, ischemic (85) and non-ischemic cardiomyopathy (86), reduce TNF-α and CRP levels, and are safe in patients irrespective of age (83, 87). The strong association between frailty and CVD and the growing database documenting safety and potential favorable effects of cell-based therapy in CVD provide justification for the assessment of potential benefits of cell therapy in subjects with frailty (88, 89; Table 1, Figure 1).

**Table 1:** The potential effects of mesenchymal stem cells (MSCs) on frailty phenotypes.

**Frailty phenotypes**	**Therapeutic MSC effects**	**Postulated mechanisms of action**
Unintentional weight loss	↓ Chronic inflammation	↓ Inflammation, ↓ Onset of sarcopenia, ↓ TNF-α, ↓ IL-6, ↓ CRP, ↓IL-1ß, ↑ IL-10, ↑ TGF-ß
Low energy levels or exhaustion	↑ Pulmonary function, ↓ Chronic inflammation	↑ Endothelial function, ↓ Biomarkers of inflammation
Weak grip strength	↑ Physical performance	↑ Endogenous stem cell function
Slow gait speed	↑ 6-min walk distance	↑ Endothelial function, ↑ Cardiac performance, ↑Skeletal muscle performance
Low physical activity	↓ Chronic inflammation, ↑ Quality of life	↓ TNF-α, ↓ IL-6, ↓ CRP, ↓IL-1ß, ↑ IL-10, ↑ TGF-ß

### Anti-inflammatory and immunomodulatory effects of mesenchymal stem cells

MSCs can evade and modulate the host's immune system to prolong their therapeutic effects without being detected and eliminated. The absence of major histocompatibility complex (MHC)/human leukocyte antigen (HLA) class II and associated costimulatory molecules and low levels of MHC/HLA class I molecules expressed by MSCs (88, 89) enables them to evade detection by the host immune system. This absence of class II molecules provides the basis for allogeneic MSC therapy, although allogeneic MSCs may eventually induce an immune reaction due to their mismatched MHC-1 molecules, which can be recognized by the host CD8+ T-cells (90).

MSCs influence the host immune system in numerous ways. They reduce both B- and T-lymphocyte proliferation in a paracrine manner (secretion of factors) and by direct cell-cell contact (91, 92). MSCs reduce the expression of proinflammatory cytokines, including, TNF-α, interleukin (IL)-1β, IL-6, and CRP [see (93–95) for review; Figure 1]. The paracrine effects of MSCs are produced in response to either secretion of a wide array of individual factors, such as growth factors and cytokines, or via exosomes, small extracellular vesicles that contain proteins, peptides and microRNAs (miRNAs).

Factors secreted by MSCs include transforming growth factor (TGF)-β, hepatocyte growth factor (HGF) and interleukins, among many others [see (93) for review]. Many of these factors interact to produce an immunomodulatory effect (96). Furthermore, the effect of a specific factor may be modulated by the microenvironment (93). Perhaps the most well studied factor secreted by MSCs is TGF-ß. MSCs produce TGF-ß in response to IL-4 receptor mediated activation of the STAT6 pathway (97). TGF-ß inhibits the proliferation of CD4+ and CD8+ T-cells and the secretion of T helper1 (Th1) cells while increasing T-regulatory cells (Treg). Another factor secreted by MSCs, IL-10, is an anti-inflammatory and immunoregulatory cytokine also expressed by a variety of immune cells. MSC expression of IL-10 requires direct interaction with T-cells (98). IL-10 inhibits the ability of macrophages to produce pro-inflammatory cytokines. However, recent evidence suggests that increased production of IL-10 by cardiac macrophages promotes diastolic dysfunction (99). This result emphasizes that the effect of a specific factor, whether secreted by MSCs or another cell type, is dependent on context and it is the combination and interaction of secreted (and resident) factors that enable MSCs to modulate the host immune system.

MSCs also affect the immune system through their release of exosomes. Exosomes are 40–100 nm extracellular vesicles that can be isolated from MSC-conditioned media. *Ex vivo* studies demonstrate that MSC-derived exosomes reduce secretion of pro-inflammatory cytokines (IL-1ß, TNF-α) and increase production of TGF-ß by PBMCs, but don't affect PBMC proliferation (100). Administration of MSCs (101, 102) or MSC-derived exosomes (103) reduces the immune response in two mouse models of autoimmune disease, Type 1 diabetes mellitus and uveoretinitis. These results and those from many other studies, suggest that MSC-derived exosomes represent an alternative to stem cell therapy.

Exosomes are secreted by many cell types, including cells of the immune system. A recent study by Ipson et al. isolated exosomes from 7 frail and 7 robust individuals who suffered from similar chronic diseases. Eight exosome-derived miRNAs were identified that were differentially expressed in these two populations and are found at higher levels in frail individuals (104). While the sample size was small, this result suggests that exosome miRNA profiles may represent biomarkers for frailty. Perhaps isolating and administering MSC-derived exosomes containing miRNAs that can counteract these “frailty-specific” miRNAs, will provide a therapeutic option for treating frailty.

Not only do MSCs affect the host's immune system, but the host immune system also modulates the activity of MSCs (105–107). Exposure to interferon (IFN)-γ generally enhances the immunosuppressive action of MSCs while simultaneously increasing their HLA class I and II cell surface marker expression (94). However, low concentrations of both IFN-γ and TNF-α, can cause MSCs to become pro-inflammatory (94, 95). Furthermore, host-derived pro-inflammatory cytokines can impair the capability of MSCs to differentiate into bone, fat, and cartilage lineages [see (94)]. However, even after MSCs have differentiated into chondrocytes, they can exert immunosuppressive effects (108, 109).

MSCs are not all equal. Recent studies suggest that the tissue from which an MSC originates influences its immunomodulatory properties. Kim et al. compared the immunosuppressive properties of MSCs isolated from periodontal ligament, umbilical cord, and adipose tissue and determined that while they all similarly inhibited the proliferation and activation of PBMCs, UC-MSCs and to a lesser extent Ad-MSCs, secreted higher levels of immunosuppressive cytokines in response to IFN-γ (110). Furthermore, MSCs obtained from aged individuals possess reduced immunomodulatory properties compared to those from the young (111, 112). The tissue microenvironment into which MSCs migrate/are injected into also influences their immunosuppressive properties. MSCs located within an inflammatory microenvironment can suppress cytotoxic T cells (96, 113), induce T regulatory cells (114, 115), and stimulate macrophage polarization (116) (transition from an M1 to an M2 phenotype) thereby promoting an anti-inflammatory milieu. Furthermore, a recent study demonstrated that MSCs exert antibacterial effects (117) indicating that MSCs possess an immune function independent of the host's immune system.

### Results of phase I and II clinical trials of MSCs for frailty

Currently, there is no specifically approved treatment for frail patients and therefore no established standard of care. The ultimate goal of a therapeutic strategy for frailty is to lengthen the healthy lifespan and restore or maintain cognitive and physical functionality of patients. We conducted a phase I and a phase II clinical trial, CRATUS (NCT02065245), investigating the safety (primary outcome) and efficacy (secondary outcome) of an intravenous infusion of allogeneic bone marrow-derived MSCs as a novel therapy for treating patients experiencing mild to moderate frailty (56, 57, 75). Efficacy outcomes included physical performance, quality of life, and systemic inflammation.

The phase I trial was a non-randomized, dose-escalation study in which 15 patients diagnosed with frailty, based on the CSHA clinical frailty scale, received allogeneic MSCs by intravenous infusion at doses of 20, 100, or 200 million MSCs (5 patients per group). All of the doses were administered as an 80 mL infusion at a speed of 2 mL/min, for a total infusion time of 40 min. Incidence of any treatment-emergent serious adverse events (TE-SAEs) at 1 month post-infusion was the primary outcome. Physical function measurements and circulating inflammatory biomarkers, measured at 3 and 6 months post-infusion, were the secondary outcomes. No TE-SAEs were reported with any of the doses at 1-month post-infusion and no clinically significant donor-specific immune reactions occurred during the first 6 months post-infusion. The six-min walk distance significantly increased at 3 and 6 months and circulating TNF-α levels significantly decreased at 6 months in all treatment groups. The best improvement in all efficacy outcomes was observed with the 100-million dose, except in the case of TNF-α, which showed a significant improvement with both the 100- and 200-million doses. The physical component of the SF-36 quality of life assessment also showed significant improvements in the 100-million dose group at all time points relative to baseline. This study indicated that allogeneic infusion of MSCs is safe and immunologically tolerated in aging frailty patients.

The phase II trial was a randomized, double-blinded, dose-finding study of intravenous allogeneic MSCs at doses of 100- or 200-million compared to placebo in 30 frailty patients (mean age 75.5 ± 7.3) (57). The primary outcome was safety, namely incidence of TE-SAEs at 1-month post-infusion. The secondary outcomes were physical performance measures, patient-reported quality outcomes, and immune markers of frailty, measured at 6 months post-infusion. There were no therapy-related TE-SAEs reported at 1-month post-infusion. Physical performance improved to a greater extent in the 100-million dose group and measures of immunologic parameters improved in both the 100-million and 200-million dose groups. The 6-min walk test, short physical performance exam, and forced expiratory volume in 1 s improved significantly in the 100-million dose group but not in the 200-million dose or placebo groups. Moreover, there was improvement noted in the female sexual quality of life questionnaire and decreases in serum TNF-α levels in the 100-million dose group. B cell intracellular TNF-α improved significantly in both the 100-million and 200-million dose groups compared to placebo. Early and late activated T-cells were decreased as well by MSC infusion compared to placebo. Although there were no safety concerns with the 200-million dose, there was no added benefit observed with this higher dose compared to the 100-million dose. In summary, intravenous allogeneic MSCs were found to be safe in individuals with aging frailty and produced significant benefits in measures of physical performance as well inflammatory biomarkers, which are important therapeutic outcomes in the frailty syndrome.

Allogeneic therapy was used in these studies because it offers “off-the-shelf” availability and a consistency to the cell product (118). These properties are extremely important as autologous cells may have deficiencies in function due to the underlying disease process, co-morbidities, lifestyle, concomitant medications, and/or patient age (119–121). Despite reports that allogeneic MSCs may be immunologically cleared more rapidly than autologous cells after differentiation (122), due to immunogenicity, this immunologic clearance might be beneficial in reducing any long-term risks of cell engraftment (123). Given the excellent safety profile and promising therapeutic efficacy demonstrated in these early phase trials (Table 1), studies with repeat dosing and longer follow up time (CRATUS; NCT02065245), as well as, a larger phase IIb (NCT03169231) clinical trial are ongoing to establish the efficacy of MSCs in the frailty syndrome.

## Author contributions

IS drafted and edited the manuscript. WB wrote a section, edited the manuscript for scientific content, and made the figure. JH edited the manuscript for scientific content.

### Conflict of interest statement

JH reported having a patent for cardiac cell-based therapy. He holds equity in Vestion Inc. and maintains a professional relationship with Vestion Inc. as a consultant and member of the Board of Directors and Scientific Advisory Board. He is the Chief Scientific Officer, a compensated consultant and advisory board member for Longeveron, and holds equity in Longeveron. He is also the co-inventor of intellectual property licensed to Longeveron. Longeveron LLC and Vestion Inc. did not participate in funding this work. The remaining authors declare that the research was conducted in the absence of any commercial or financial relationships that could be construed as a potential conflict of interest.
